# Clinical factors associated with treatment outcomes in EGFR mutant non-small cell lung cancer patients with brain metastases: a case-control observational study

**DOI:** 10.1186/s12885-019-6140-0

**Published:** 2019-10-26

**Authors:** Yung-Hsuan Chen, Yen-Fu Chen, Chung-Yu Chen, Jin-Yuan Shih, Chong-Jen Yu

**Affiliations:** 1 Division of Pulmonary and Critical Care Medicine, Department of Internal Medicine, National Taiwan University Hospital Yunlin Branch, No.579, Sec. 2, Yunlin Rd., Douliu City, Yunlin County 640 Taiwan, Republic of China; 2 Division of Pulmonary and Critical Care Medicine, Department of Internal Medicine, National Taiwan University Hospital, College of Medicine, National Taiwan University, No.7, Chung Shan S. Rd., Taipei City, 100 Taiwan, Republic of China

**Keywords:** NSCLC, EGFR-TKI, Brain metastasis, Brain radiotherapy, Uncommon mutations

## Abstract

**Background:**

Non-small cell lung cancer (NSCLC) patients harboring epidermal growth factor receptor (EGFR) mutations often develop brain metastases. Treatment with EGFR-tyrosine kinase inhibitors (TKIs) has shown the effectiveness; however, knowledge of the clinical factors associated with outcomes in NSCLC patients with EGFR mutations remains limited.

**Methods:**

Treatment-naive patients diagnosed with advanced non-squamous NSCLC with brain metastases harboring EGFR mutations and treated with an EGFR-TKI as first-line therapy were enrolled with analysis of their medical records.

**Results:**

A total of 134 advanced NSCLC patients with brain metastases harboring EGFR mutations received an EGFR-TKI (gefitinib: 62, erlotinib: 49, and afatinib: 23) as the first-line therapy. Sixty-nine had exon 19 deletions (51.5%), and 56 (41.8%) had L858R mutations. There was no statistically significant difference in progression-free survival (PFS) and overall survival (OS) among the EGFR-TKIs. Significantly shorter OS was noted in patients with multiple brain metastases (hazard ratio [HR]: 2.43, *p* = 0.007), uncommon EGFR mutations (HR: 3.75, *p* = 0.009), and liver metastases. Thirty-eight patients (29.1%) received brain radiotherapy for brain metastases before disease progression, and had a significantly longer time until intracranial progression. However, the brain radiotherapy had no statistically significant impact on PFS or OS.

**Conclusions:**

Patients with uncommon mutations, multiple brain metastases, and concomitant liver metastases tended to have shorter OS. Brain radiotherapy could delay the time to intracranial disease progression but had no impact on survival. The different first-line EGFR-TKIs achieved similar treatment responses in terms of PFS and OS in the EGFR-mutated NSCLC patients with brain metastases.

## Background

Of patients with non-small cell lung cancer (NSCLC), approximately 25 to 40% develop brain metastases (BM), [1] with adenocarcinomas accounting for more than half of all NSCLC BM [2]. Even when treated with whole brain radiotherapy (WBRT), patients with BM have typically had poor prognoses in the past, including a median survival of only around 6 months [3]. Aside from WBRT, NSCLC patients with BM have shown some responsiveness to chemotherapy with pemetrexed or cisplatin combined with other types of chemotherapy, including response rates ranging from 27 to 69% and overall survival (OS) durations ranging from 7.4 to 10 months [4–6]. In recent years, randomized trials have further reported that EGFR-tyrosine kinase inhibitors (TKIs) have shown better progression-free survival (PFS), objective responses, and safety profiles than standard first-line platinum-based doublet chemotherapy in patients with positive EGFR-mutant NSCLC, such that EGFR-TKIs have become the standard treatment for the initial management of EGFR-mutant advanced NSCLC [7–10]. Relatedly, some studies have reported encouraging results for the treatment of positive EGFR-mutant NSCLC patients with BM with EGFR-TKIs alone, including PFS durations of 6.6 to 15.2 months and OS durations of 12.9 to 18.9 months [11–15].

Among the EGFR-TKIs, the first-generation EGFR-TKIs gefitinib and erlotinib are inherently different in their method of action from the second-generation EGFR-TKI afatinib, with the former reversibly binding to cause the inhibition of EGFR signaling and the latter irreversibly blocking the ErbB family of receptors. Data collected in previous research has further shown that these EGFR-TKIs have different in vitro sensitivities, different plasma drug concentrations, and different clinical responses to TKIs [16–18]. Meanwhile, given the lack of any direct comparisons of these drugs via prospective randomized trials, a series of meta-analyses were conducted in order to determine which EGFR-TKI, if any, is the most effective. These studies, however, did not find any significant differences in the effectiveness of afatinib, erlotinib, and gefitinib [19–21]. Furthermore, the role played by TKIs in patients with BM is still not clear.

As with the use of EGFR-TKIs, the survival impacts of other local treatments for BM, such as surgical tumor excision and radiotherapy, have also not been thoroughly clarified. As such, we conducted the present study in order to provide a clearer picture of the effects both of various clinical factors and different TKIs in BM patients with EGFR-activating mutations. To that end, we sought to determine the prognostic factors for survival via a retrospective analysis of the clinical impacts of BM number and other metastatic locations, EGFR mutation type, and additional treatments (specifically, surgical excision or radiotherapy) for BM. At the same time, the analysis also allowed us to assess the respective treatment efficacies of afatinib, erlotinib, and gefitinib in EGFR-mutant NSCLC patients with BM.

## Methods

### Patient cohort

The investigation was approved by the National Taiwan University Hospital (NTUH) Research Ethics Committee. In this retrospective study, patients aged 18 years or older with non-squamous NSCLC who had ever received an EGFR-TKI as the first-line treatment during the period from May 1, 2013, to May 31, 2016, at NTUH or NTUH Yunlin Branch were identified. The patients could be newly diagnosed with non-squamous NSCLC at either of those two hospitals or referred from other hospitals. EGFR gene mutation detection was measured by MassARRAY genotyping (SEQUENOM). The exclusion criteria included the following: patients without an EGFR-activating mutation (such as an L858R mutation in exon 21 or an exon 19 deletion) or another uncommon mutation, patients who received treatment for less than 3 months because of adverse effects or other comorbidities, patients who were lost to follow-up within 3 months, and patients for whom there was incomplete data for analysis.

The medical records data of each patient, including age at diagnosis, gender, smoking history, comorbidities, EGFR mutation type, BM number, other metastatic locations, and treatment modalities were retrospectively reviewed and recorded. Chest computed tomography (CT), brain imaging (CT or magnetic resonance imaging (MRI)), and bone scans were undertaken for initial staging. The patients took an EGFR-TKI (gefitinib 250 mg/day, erlotinib 100 mg or 150 mg/day, or afatinib 30 mg or 40 mg/day), received other treatments for BM (radiotherapy or surgical excision), and obtained subsequent anticancer therapy after disease progression according to their physicians’ instructions. Follow-up imaging was arranged every 3 months after TKI treatment or as needed at the physicians’ discretion to confirm the treatment response. The treatment responses were evaluated according to the Response Evaluation Criteria in Solid Tumors version 1.1 [22] and defined as complete remission (CR), partial response (PR), stable disease (SD), or progressive disease (PD). The proportion of patients who had CR or a PR to therapy was defined as the overall response rate (ORR). The intracranial responses were also recorded according to the above criteria.

Patients were enrolled for PFS and OS analysis. Information on survival was obtained through active follow-up based on verification of each patient’s vital status. PFS was defined as the duration from the beginning of EGFR-TKI treatment until the time of disease progression. OS was defined as the period from the date of beginning EGFR-TKI treatment to the date of death or the last follow-up.

### Statistical analysis

Continuous variables are expressed as medians with ranges, and categorical variables are expressed as percentages of the group from which they were derived. Categorical variables were compared using the Chi-square test. Kaplan-Meier curves were plotted for OS, PFS, and the subgroups of clinical factors, and the log-rank test was used to determine statistical significance. Cox proportional-hazards regression was used for covariate analysis to determine the hazard ratio of clinical factors and survival. A *p* value less than 0.05 was considered significant, and factors with a *p* value ≤0.01 were added to a multivariate Cox regression model. All analyses were performed using MedCalc Statistical Software version 18.5 (MedCalc Software bvba, Ostend, Belgium; http://www.medcalc.org; 2018). The data cut-off date was December 31, 2017.

## Results

### Patient characteristics

**Fig. 1:**
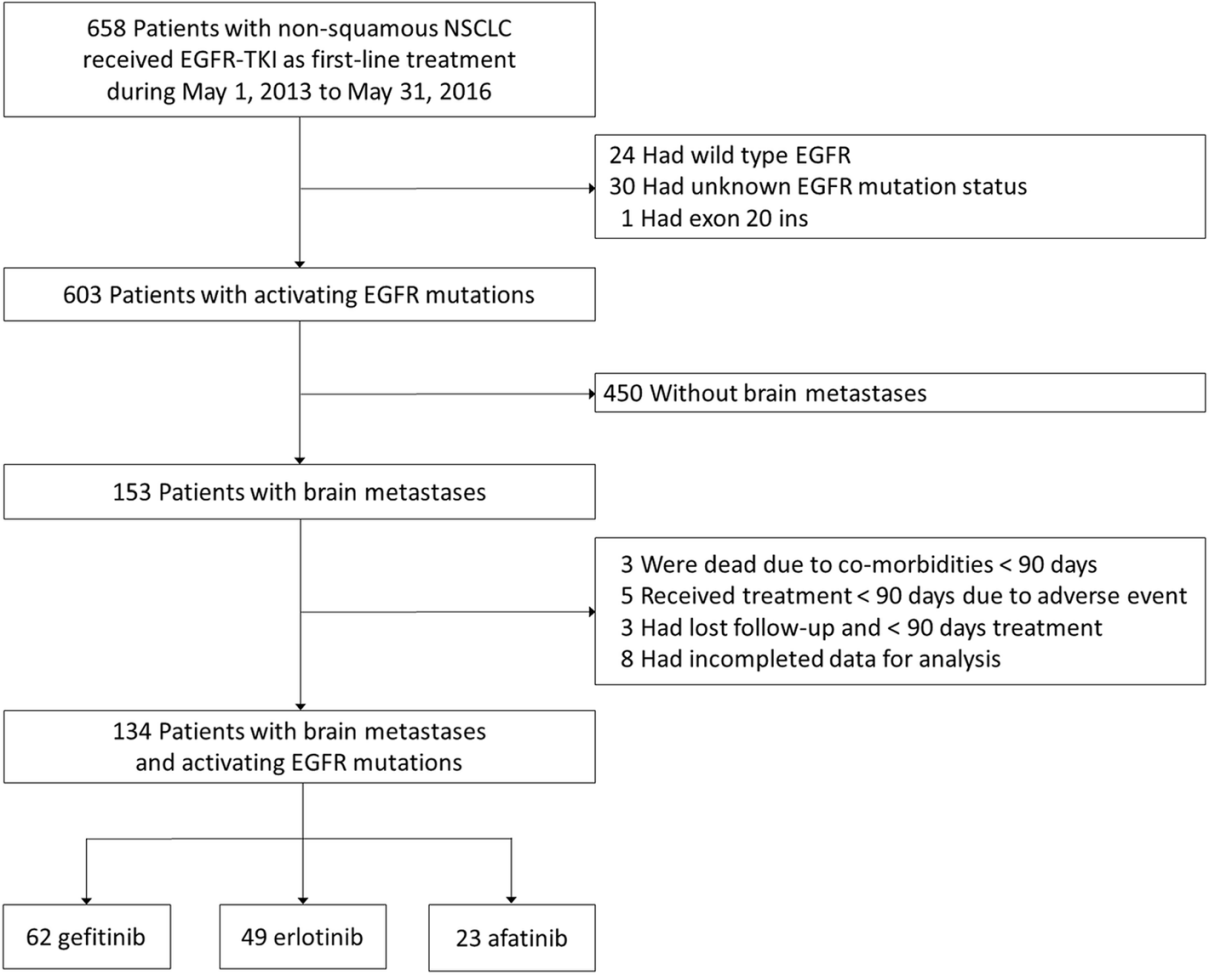
Patient selection and exclusion criteria

From May 1, 2013, to May 31, 2016, 658 patients with stage IIIB or IV lung cancer received an EGFR-TKI as first-line therapy. After excluding those who met the exclusion criteria, a total of 134 patients were enrolled in the study (Fig. 1). Sixty-two patients received gefitinib, 49 patients received erlotinib, and 23 patients received afatinib (Table 1). Ninety-six patients were female (71.6%). Only 16 patients were smokers (11.9%). There were 70 patients (52.2%) who underwent brain MRI to confirm BM at the initial staging, while the other 64 patients underwent brain CT to confirm BM at the initial staging. There were 56 patients who harbored L858R mutations (41.8%), 69 patients who harbored exon 19 deletions (51.5%), 5 patients who harbored uncommon mutations (2 with G719X and 3 with G719A), and 4 patients who harbored complex mutations (3 with L858R + T790 M and another 1 with L858R + S768I). Of the 134 patients, 123 patients (91.8%) had multiple distant metastases (M1c) [23], with the largest number of patients having bone metastases (*n* = 87, 64.9%) and the next largest number of the patients having liver metastases (*n* = 26, 19.4%). Eighty-eight patients (65.7%) had three or more BM. Among the 38 patients who received radiotherapy to treat BM following the confirmation of BM, 22 patients (57.9%) exhibited neurological symptoms. Only 8 patients (6.0%) received brain tumor excision at the beginning of their treatment courses, including 2 patients who did not exhibit neurological symptoms.

**Table 1:** Basic characteristics of 134 non-small cell lung cancer patients with brain metastases

Characteristics	All (*N* = 134)	Gefitinib (*N* = 62)	Erlotinib (*N* = 49)	Afatinib (*N* = 23)	*p* value
Age > 65 years old	61 (45.5%)	29 (46.8%) (38–93 yr)	26 (53.1%) (41–86 yr)	6 (26.1%) (42–81 yr)	.097
Male sex	38 (28.4%)	17 (27.4%)	13 (26.5%)	8 (34.8%)	.750
Smoking	16 (11.9%)	8 (12.9%)	4 (8.2%)	4 (17.4%)	.504
EGFR mutation status					.046
L858R	56 (41.8%)	26 (41.9%)	26 (53.1%)	4 (17.4%)	
Del 19	69 (51.5%)	31 (50.0%)	21 (42.9%)	17 (74.0%)	
Uncommon mutation	5 (3.7%)	3 (4.8%)	2 (4.1%)	0	
Complex mutations	4 (3.0%)	2 (3.2%)	0	2 (8.7%)	
Initial brain metastases number ≥ 3	88 (65.7%)	33 (53.2%)	33 (67.3%)	15 (65.2%)	.527
Other metastastic location					
Lung	54 (40.3%)	24 (38.7%)	21 (42.9%)	9 (39.1%)	.900
Bone	87 (64.9%)	42 (67.7%)	32 (65.3%)	13 (56.5%)	.627
Liver	26 (19.4%)	8 (12.9%)	12 (24.5%)	6 (26.1%)	.208
Pleura	35 (26.1%)	21 (33.9%)	8 (16.3%)	6 (26.1%)	.113
Other location	23 (17.2%)	5 (8.1%)	12 (24.5%)	6 (26.1%)	.034
M1c (definition by AJCC 8th edition)	123 (91.8%)	56 (90.3%)	48 (98.0%)	19 (82.6%)	.073
Radiotherapy to brain metastases	38 (29.1%)	24 (38.7%)	13 (26.5%)	2 (8.7%)	.018
Brain tumor excision	8 (6.0%)	7 (11.3%)	1 (2.0%)	0	.052

The ORR to the various EGFR-TKIs was 74.6%, and the respective response rates to gefitinib, erlotinib, and afatinib were 79.4, 69.4, and 73.9% (Table 2). The most common adverse effects (≥ grade 2) were skin rash or itching (*n* = 4, 3.0%), paronychia (*n* = 2, 1.5%), and diarrhea (*n* = 1, 0.7%). Patients who received afatinib experienced more adverse effects and also had a higher rate of paronychia (4.3%) than those who received gefitinib or erlotinib (*p* = 0.005). Other critical drug-related adverse effects such as pneumonitis or hepatitis were not mentioned.

**Table 2:** Treatment responses of 134 non-small cell lung cancer patients with brain metastases

	All (*N* = 134)	Gefitinib (*N* = 62)	Erlotinib (*N* = 49)	Afatinib (*N* = 23)	*p* value
Treatment response					.053
PR	100 (74.6%)	49 (79.4%)	34 (69.4%)	17 (73.9%)	
SD	26 (19.4%)	12 (19.0%)	12 (24.5%)	2 (8.7%)	
PD	8 (6.0%)	1 (1.6%)	3 (6.1%)	4 (17.4%)	
Intracranial response					.208
CR	56 (41.8%)	21 (33.9%)	23 (46.9%)	12 (52.2%)	
PR	26 (19.4%)	12 (19.4%)	11 (22.4%)	3 (13.0%)	
SD	48 (35.8%)	28 (45.2%)	14 (28.6%)	6 (26.1%)	
PD	4 (3.0%)	1 (1.6%)	1 (2.0%)	2 (8.7%)	
PD location					.055
Intracranial only	32 (23.9%)	16 (27.0%)	10 (20.4%)	6 (26.1%)	
Extracranial only	73 (54.5%)	40 (63.5%)	22 (44.9%)	11 (47.8%)	
Both	13 (9.7%)	4 (6.3%)	6 (12.2%)	3 (13.0%)	
Common adverse effects (≥ grade 2)					
Skin rash or itching	4 (3.0%)	1 (1.6%)	1 (2.0%)	2 (8.7%)	.164
Diarrhea	1 (0.7%)	0 (0.0%)	0 (0.0%)	1 (4.3%)	.115
Paronychia	2 (1.5%)	0 (0.0%)	1 (2.0%)	1 (4.3%)	.005
Median PFS (months) [95% CI]	11.4 [9.30 to 13.30]	12.1 [9.00 to 14.50]	10.6 [8.80 to 40.60]	10.4 [7.50 to 17.20]	.783^a^
Time to intracranial PD (months) [95% CI]	23.6 [17.20 to 30.10]	23.6 [17.00 to 30.10]	27.8 [11.30 to 27.80]	17.2 [10.40 to 19.00]	.729^a^
Median OS (months) [95% CI)	36.9 [29.10 to 60.00]	38.2 [29.10 to 60.00]	NA^b^	29.6 [24.80 to 33.00]	.695^a^

^a^Log-rank test

^b^The median overall survival (OS) of the erlotinib group could not be computed. Instead, the median OS of the erlotinib group was 36.9 months (95% CI 19.90 to 36.90) if the Kaplan-Meier survival curves for 60 months were calculated

### Clinical factors associated with survival of NSCLC patients with BM

Disease progression had occurred in 118 patients by the end of the follow-up period (88.1%), and 45 patients had intracranial progression (33.6%), including 13 patients who had both intracranial and extracranial progression (Table 2).

**Fig. 2:**
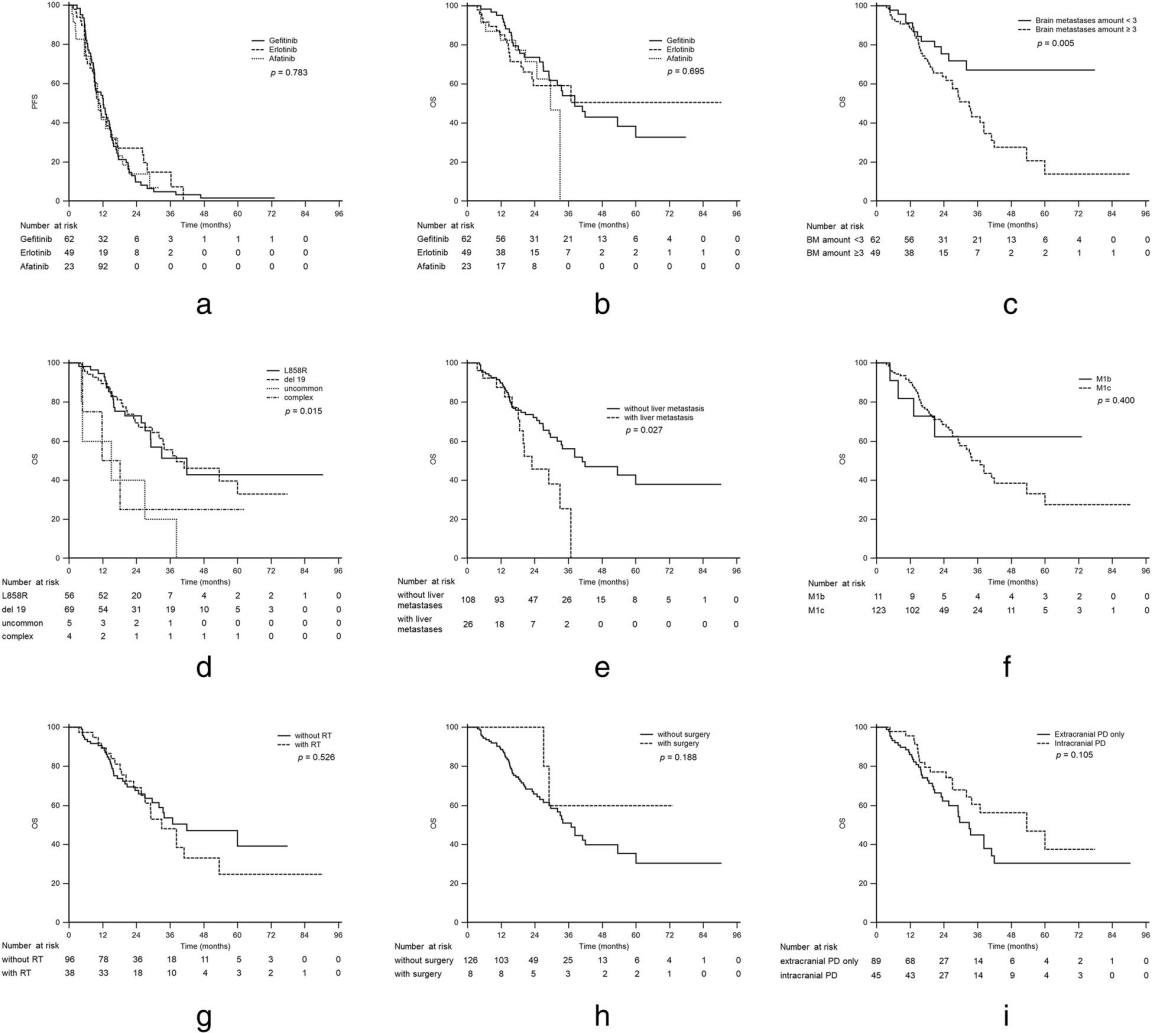
Progression-free survival (**a**) and overall survival (**b**) in patients with brain metastases treated with gefitinib, erlotinib, or afatinib; overall survival in patients with different numbers of brain metastases (**c**), different epidermal growth factor receptor gene mutation types (**d**), concomitant liver metastases (**e**), and different M stages (**f**); overall survival in patients with and without radiotherapy (**g**) and with and without surgical excision (**h**); and overall survival in patients with different locations of disease progression (**i**)

The median PFS and OS for all the patients were 11.4 (95% CI: 9.30 to 13.30) and 36.9 (95% CI: 29.10 to 60.00) months, respectively. There were no statistically significant differences in PFS and OS among the three EGFR-TKIs (Table 2, Fig. 2a and b), nor were there statistically significant differences in PFS and OS for other clinical factors such as age and smoking status (Table 3).

**Table 3:** Cox proportional hazards regression of all patients for progression-free survival and overall survival

Variables	Progression-free survival		Overall survival	
	Hazard ratio (95% CI)	*p* value	Hazard ratio (95% CI)	*p* value
Age > 65	1.11 (0.763 to 1.604)	0.594	1.11 (0.653 to 1.894)	0.694
Sex (male)	0.94 (0.628 to 1.422)	0.786	1.38 (0.787 to 2.407)	0.263
Smoking	1.08 (0.605 to 1.936)	0.791	1.68 (0.820 to 3.450)	0.156
EGFR mutation				
L858R	Reference		Reference	
Del 19	0.97 (0.664 to 1.413)	0.867	0.99 (0.558 to 1.772)	0.985
Uncommon mutation	2.40 (0.851 to 6.753)	0.098	3.75 (1.401 to 10.027)	0.009
Complex mutations	3.12 (1.111 to 8.777)	0.038	2.20 (0.645 to 7.523)	0.208
Initial brain metastases number ≥ 3	1.17 (0.796 to 1.726)	0.422	2.43 (1.277 to 4.629)	0.007
Other metastases location				
Lung	1.33 (0.921 to 1.918)	0.129	1.56 (0.925 to 2.642)	0.095
Bone	1.42 (0.963 to 2.090)	0.077	1.70 (0.948 to 3.045)	0.075
Liver	1.58 (0.981 to 2.558)	0.060	2.03 (1.071 to 3.831)	0.041
Pleura	1.33 (0.880 to 2.019)	0.175	1.42 (0.808 to 2.495)	0.223
Other location	1.84 (1.112 to 3.038)	0.018	1.59 (0.818 to 3.085)	0.172
M1c (definition by AJCC 8th edition)	1.63 (0.812 to 3.269)	0.169	1.55 (0.555 to 4.348)	0.402
Radiotherapy to brain metastases	0.78 (0.523 to 1.175)	0.239	1.19 (0.690 to 2.064)	0.527
Brain tumor excision	0.75 (0.344 to 1.630)	0.466	0.40 (0.097 to 1.645)	0.204
PD location				
Intracranial only	Reference		Reference	
Extracranial only	1.10 (0.724 to 1.683)	0.646	1.71 (0.903 to 3.259)	0.100
Both	1.03 (0.537 to 1.978)	0.927	0.90 (0.322 to 2.541)	0.849
TKI				
Gefitinib	Reference		Reference	
Erlotinib	0.88 (0.584 to 1.324)	0.539	1.14 (0.627 to 2.065)	0.670
Afatinib	1.03 (0.620 to 1715)	0.907	1.39 (0.642 to 3.001)	0.404

**Fig. 3:**
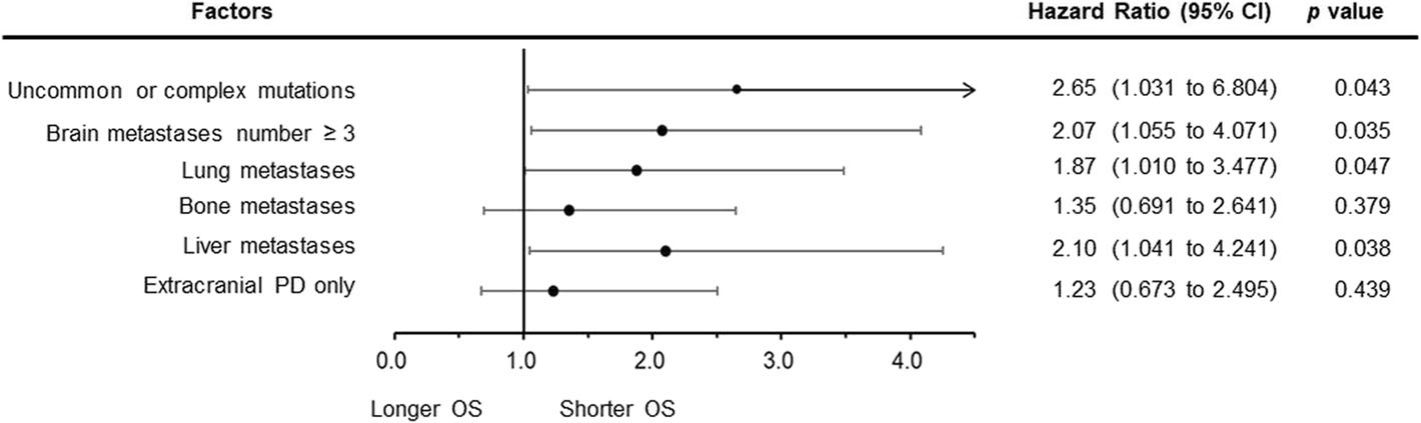
Multivariate Cox regression analysis of overall survival in NSCLC patients with brain metastases

Significantly shorter OS was noted in the patients with multiple BM (median: 33.0 months, 95% CI: 24.80 to 38.20, *p* for log-rank test = 0.005, Fig. 2c; Table 3), uncommon EGFR mutations and complex mutations (L858R vs. del 19 vs. uncommon vs. complex: 41.9 vs. 38.2 vs. 15.0 vs. 11.7 months, respectively, *p* for log-rank test = 0.015, Fig. 2d; Table 3), and liver metastases (median: 23.0 months, 95% CI: 18.20 to 36.90, *p* for log-rank test = 0.027, Fig. 2e; Table 3). Patients with multiple extrathoracic metastases in one or more organs, which is known as M1c disease according to the eighth edition of the TNM staging system [23], had shorter median OS, but the difference was not statistically significant (36.9 months, 95% CI: 29.00 to 53.50, *p* for log-rank test = 0.400, Fig. 2f; Table 3). Patients harboring uncommon mutations and complex mutations had significantly shorter PFS than those with common mutations (Additional file 1: Figure S1, median: L858R vs. del 19 vs. uncommon vs. complex: 12.3 vs. 11.4 vs. 9.3 vs. 5.8 months, respectively, *p* = 0.040). A multivariate analysis by Cox regression model showed that uncommon or complex EGFR mutations (HR: 2.65, 95% CI: 1.031 to 6.804, *p* = 0.043), multiple BM (HR: 2.07, 95% CI: 1.055 to 4.071, *p* = 0.035), and concomitant liver metastases were poor prognostic factors in terms of OS (Fig. 3).

The additional local treatment (radiotherapy or tumor excision) for BM had no statistically significant impact on PFS or OS (median OS of patients who received radiotherapy: 33.0 months, 95% CI: 25.70 to 53.50, *p* for log-rank test = 0.526, Fig. 2g; median OS of patients who did not receive tumor excision: 36.9 months, 95% CI: 29.10 to 53.50, *p* for log-rank test = 0.188, Fig. 2h; Table 3). Patients with intracranial progression had longer OS than those with only extracranial progression, but the difference was not statistically significant (median: 53.5 months, 95% CI: 32.00 to 60.00, *p* for log-rank test = 0.101, Fig. 2i).

### Evaluation of metastatic brain lesions

There were 38 patients (29.1%) who received radiotherapy for BM before disease progression. Fewer NSCLC patients with BM who were treated with afatinib initially received brain radiotherapy (2/23, 8.7%, *p* = 0.018) (Table 1). The overall intracranial treatment response rate to the various EGFR-TKIs was 61.2% (Table 2). Afatinib and erlotinib had higher intracranial response rates than gefitinib (afatinib: 65.2%, erlotinib: 69.4%, and gefitinib: 53.2%, respectively) (Table 2).

The median times to intracranial PD for gefitinib, erlotinib, and afatinib were 23.6, 27.8, and 17.2 months, respectively, but the differences in these median times to intracranial PD were not significant (Table 2). In addition, there was no statistically significant difference in time to intracranial PD for other clinical factors such as gender, age, smoking status, or number of BM (Table 4).

**Table 4:** Cox proportional hazards regression of all patients for intracranial progression

Variables	Hazard ratio (95% CI)	*p* value
Age > 65	1.12 (0.771 to 1.615)	0.561
Sex (male)	0.93 (0.617 to 1.395)	0.718
Smoking	1.06 (0.594 to 1.900)	0.838
EGFR mutation		
L858R only	Reference	
Del 19 only	0.94 (0.643 to 1.363)	0.729
Uncommon mutation	2.26 (0.802 to 6.341)	0.123
Complex mutations	2.95 (1.050 to 8.264)	0.040
Initial brain metastases number ≥ 3	1.16 (0.800 to 1.685)	0.432
RT to brain before 1st PD	0.80 (0.535 to 1.191)	0.270
Brain tumor excision	0.91 (0.608 to 1.362)	0.209
TKI		
Gefitinib	Reference	
Erlotinib	0.85 (0.568 to 1.283)	0.447
Afatinib	1.00 (0.602 to 1.662)	1.000

**Fig. 4:**
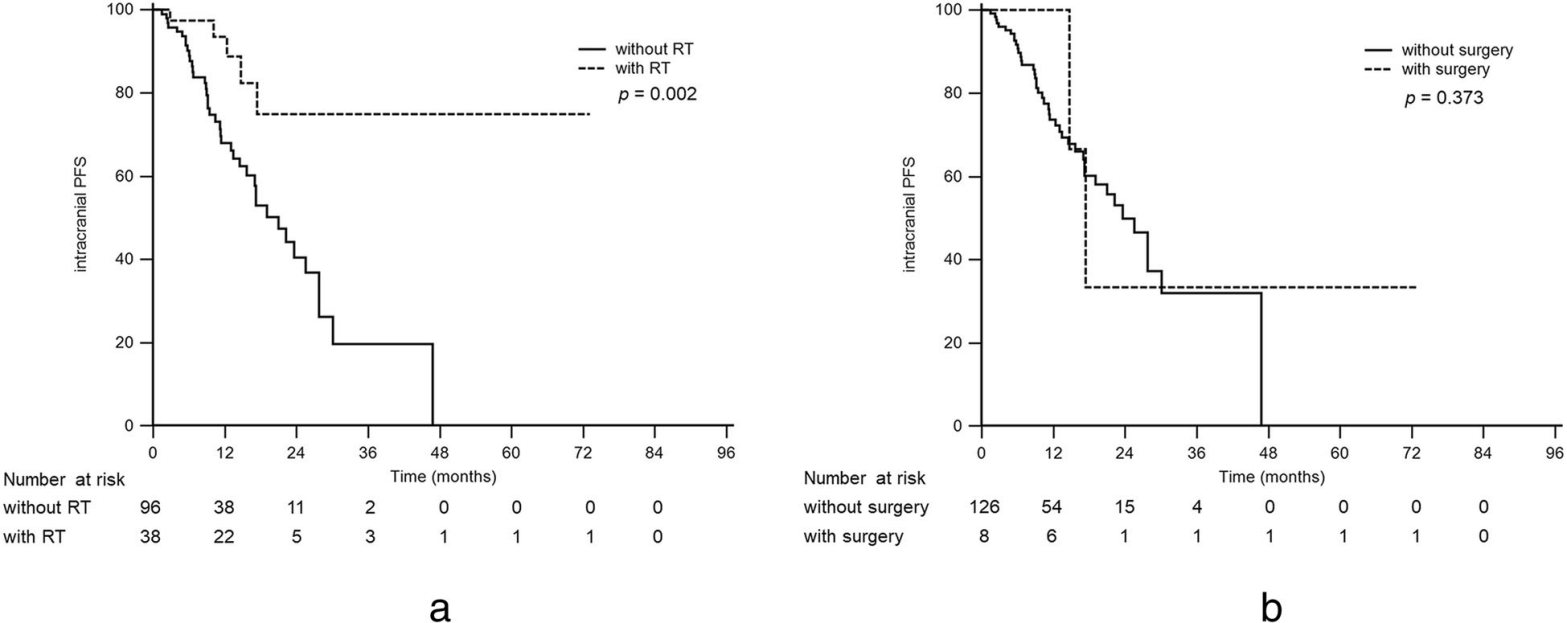
Intracranial progression-free survival in patients with and without radiotherapy (**a**) and with and without surgical excision (**b**)

Patients who received additional brain radiotherapy for BM at the initial diagnosis had a significantly longer time to intracranial PD (median time to intracranial PD, received brain radiotherapy vs. without brain radiotherapy, NR [not reached] vs. 21.0 months, *p* = 0.002, Fig. 4a). However, the additional surgical tumor excision for BM resulted in no statistically significant extension of the time to intracranial PD (median time to intracranial PD, received surgical excision vs. without surgical excision, 17.4 vs. 23.6 months, *p* = 0.373, Fig. 4b).

## Discussion

In this retrospective study, we found there was no statistically significant difference in PFS or OS among the three EGFR-TKIs in the real-world setting of the investigated patients. Meanwhile, it was found that sensitizing rare mutations, multiple BM, and concomitant with liver metastases could be independent prognostic factors for survival. Furthermore, the patients with extracranial progression and M1c disease had shorter OS, but the differences between the groups were not statistically significant. Additional brain radiotherapy for BM during the use of EGFR-TKIs showed a benefit in terms of the time to intracranial PD; however, the brain radiotherapy had no statistically significant impact on survival itself.

In real-world settings, the initial assessment of cancer status might have a greater impact on a patient’s prognosis than the different treatments available. Older age, poor performance status, extracranial metastases, and more BM have previously been found to indicate a poor prognosis in terms of estimating the survival of patients with BM [24]. A recent update to that research suggested that two additional factors, namely, EGFR and ALK alterations in patients with lung adenocarcinoma, can be used to better evaluate the prognosis of such patients [25]. In our study, patients with three or more BM and with uncommon or complex mutations were associated with shorter OS. Among the different sites of extracranial metastases, concomitant liver metastases were also associated with shorter OS in our analysis. This finding was consistent with the fact that liver metastases have also been observed as a predictor of poorer prognosis in a few retrospective studies despite the patients in those studies receiving different first-line treatments [26, 27].

About 10% patients with NSCLC harbor uncommon mutations [28]. Uncommon EGFR mutations have been found to constitute a distinct part of the whole group of EGFR mutations and to have different reactions to EGFR-TKIs [29]. The post-hoc analysis of the NEJ002 study demonstrated shorter OS for gefitinib-treated patients with uncommon mutations compared to those with common mutations (11.9 versus 29.3 months; *p* < 0.001) [30]. Nevertheless, afatinib seemed effective in patients with certain types of uncommon EGFR mutations, including G719, L861Q, and S768I, in a post-hoc study of the LUX-Lung 2, LUX-Lung 3, and LUX-Lung 6 studies (median OS 19.4 months, 95% CI: 16.4–26.9) [28]. In our study, 5 patients harbored uncommon mutations with G719, but none of those patients received afatinib treatment. Those patients had significantly shorter OS compared to those with common mutations. The effectiveness of different TKIs in NSCLC patients with uncommon EGFR mutations still needs to be further investigated.

Previous studies have described the efficacy of erlotinib in BM and attributed its efficacy to its ability to cross the blood-brain barrier [14, 31]. Meanwhile, gefitinib has been reported to be less effective than erlotinib in treating BM because of insufficient levels of the drug in the CSF (cerebrospinal fluid) [32]. Thus, some physicians may prefer to use erlotinib to treat BM before the use of afatinib has been validated. Nevertheless, one retrospective study found that BM at initial diagnosis had no impact on OS in EGFR mutation-positive patients treated with first-line gefitinib [26]. A subgroup analysis of the LUX-Lung 3 and LUX-Lung 6 studies disclosed that afatinib significantly improved the PFS (8.2 versus 5.4 months; HR, 0.50; *p* = 0.0297) and objective response rate versus chemotherapy in patients with BM [33]. Another study also showed that afatinib was effective against central nervous system metastases in heavily pretreated patients with EGFR-mutated or EGFR–TKI-sensitive NSCLC [15]. In our study, we sought to compare three EGFR-TKIs in treating BM. There was no significant difference in either PFS or OS or the development of intracranial progression in real-world practice. Unlike in the previous study, intracranial progression developed sooner (median: 8.9 months, 95% CI: 9.10 to 14.93) in our patients treated with erlotinib, but the difference was not significant. The gefitinib-treated patients had the longest median PFS and OS among the three groups, although the differences were not significant. In this retrospective analysis, the afatinib group had less patients than the gefitinib and erlotinib groups because afatinib was a newly licensed drug in Taiwan at that time. Nonetheless, this selection bias might have caused its effect to be underestimated because of the relatively short observation period. Osimertinib, a third-generation EGFR-TKI, showed longer median PFS in untreated EGFR-mutated advanced NSCLC patients with BM (osimertinib vs. standard EGFR-TKI: 15.2 vs 9.6 months, *p* < 0.001) in the phase III FLAURA trial [10]. In Taiwan, osimertinib has only been approved for second-line treatment for advanced NSCLC patients who harbor a T790 M mutation since November 2016, and reimbursements for it are not provided by the National Health Insurance system. The real-world experiences of patients treated with osimertinib compared to other EGFR-TKIs thus require more study.

Our study demonstrated that brain radiotherapy could prolong the time to intracranial PD significantly. However, additional local treatment for BM, whether radiotherapy or surgical excision, had no impact on the PFS or OS of those patients. Previous studies had shown good safety and favorable objective response rates or improvements in quality of life in patients receiving combination therapy with gefitinib [34] or erlotinib [35] but no statistically significant differences in OS. The prevention of intracranial progression seemed not to have an impact on OS. Twelve patients in this study received radiotherapy for BM earlier than they took EGFR-TKIs (median duration between radiotherapy and the beginning of EGFR-TKI use: 12.5 days, Additional file 2: Table S1). Their PFS and OS were not significantly different than those of the other 27 patients who started radiotherapy after taking EGFR-TKIs (median duration between EGFR-TKI use and the beginning of radiotherapy: 11.6 days). Eight patients in our study received surgical excision of BM. Two of them received the surgery after they started taking EGFR-TKIs for better local control because of large brain tumors (5.6 cm and 6.4 cm in diameter, respectively). Both of those patients received EGFR-TKIs for nearly 7 weeks after the surgery and had only extracranial progression later with significantly shorter PFS than the other 6 patients (Additional file 3: Table S2). This observation might indicate the importance of early systemic treatment in BM patients to prevent further extracranial progression.

This study had several limitations. First, the number of patients who received afatinib was relatively small compared to the numbers of patients who received the other two EGFR-TKIs. We enrolled patients who received an EGFR-TKI as first-line treatment from May 2013 to May 2016. Compared to gefitinib and erlotinib, which were reimbursed for first-line treatment of advanced NSCLC with EGFR mutations by the National Health Insurance system of Taiwan beginning on June 2011 and November 2013, respectively, afatinib was only reimbursed beginning in May 2014. The relatively late licensing of afatinib may have impacted the number of patients treated with the drug in our study. Length bias may also have affected the study. Second, based on the current evidence, the existence of BM [36] and different EGFR mutation types [37] may affect physicians’ decisions in terms of prescribing EGFR-TKIs. Third, the numbers of BM in patients and the number of patients with intracranial progression may have been underestimated in those patients who only received brain CT during the initial staging or follow-up period.

## Conclusion

In EGFR-mutant NSCLC patients with BM, uncommon or complex mutations, multiple BM, and concomitant liver metastases tended to have shorter OS. Brain radiotherapy could be considered for early symptomatic BM patients to improve the time to intracranial PD; however, the intervention had no statistically significant impact on survival. In clinical practice, the difference among the three EGFR-TKIs on PFS and OS was not significant. An advanced prospective randomized control trial would be warranted to compare the clinical efficacy between first- and second-generation EGFR-TKI treatments for EGFR-mutant NSCLC patients with BM.

## Supplementary information

**Additional file 1: Figure S1.** Progression-free survival in patients with different epidermal growth factor receptor gene mutation types.

**Additional file 2: Table S1.** Comparison of treatment responses in patients receiving radiotherapy before and after tyrosine kinase inhibitor usage.

**Additional file 3: Table S2.** Comparison of surgery responses in patients receiving surgery before and after tyrosine kinase inhibitor usage. *The median overall survival (OS) could not be computed.

## Data Availability

All data generated or analyzed during this study are included in this published article.

## Abbreviations

BM: Brain metastases
CI: Confidence interval
CR: Complete remission
CT: Computed-tomography
EGFR: Epidermal growth factor receptor
HR: Hazard rate
NSCLC: Non-small cell lung cancer
OS: Overall survival
PD: Progressive disease
PFS: Progression-free survival
PR: Partial response
SD: Stable disease
TKI: Tyrosine kinase inhibitors
WBRT: Whole brain radiotherapy
