# Supplementary figures and images for: Clinical factors associated with treatment outcomes in EGFR mutant non-small cell lung cancer patients with brain metastases: a case-control observational study
Source: BMC Cancer. 2019 Oct 26;19:1006. doi: 10.1186/s12885-019-6140-0 (PMC6815404; doi:10.1186/s12885-019-6140-0)

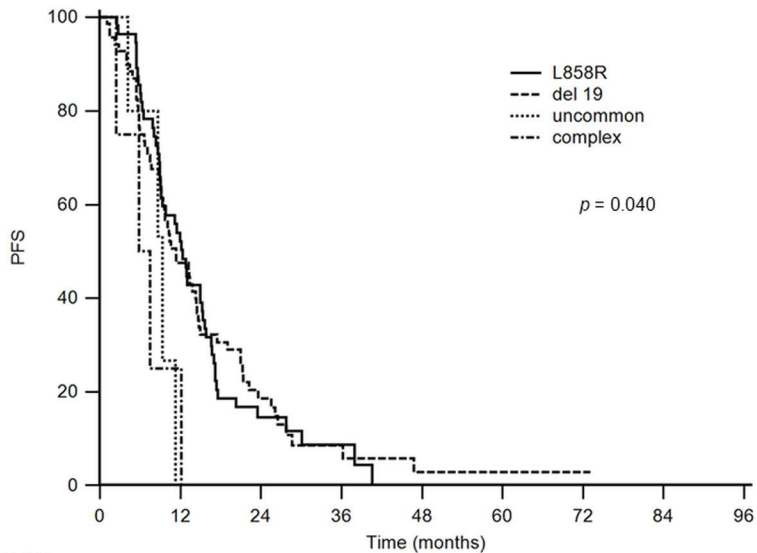

Number at risk

|          |    |    |    |   |   |   |   |   |
|----------|----|----|----|---|---|---|---|---|
| L858R    | 56 | 28 | 6  | 2 | 0 | 0 | 0 | 0 |
| del 19   | 69 | 31 | 10 | 3 | 1 | 1 | 1 | 0 |
| uncommon | 5  | 0  | 0  | 0 | 0 | 0 | 0 | 0 |
| complex  | 4  | 1  | 0  | 0 | 0 | 0 | 0 | 0 |

Supplement: Supplementary file 1 — Additional file 1: Figure S1. Progression-free survival in patients with different epidermal growth factor receptor gene mutation types. [file 12885_2019_6140_MOESM1_ESM.pdf]
