# Supplementary material for: Clinical factors associated with treatment outcomes in EGFR mutant non-small cell lung cancer patients with brain metastases: a case-control observational study
Source: BMC Cancer. 2019 Oct 26;19:1006. doi: 10.1186/s12885-019-6140-0 (PMC6815404; doi:10.1186/s12885-019-6140-0)
Supplement: Supplementary file 2 — Additional file 2: Table S1. Comparison of treatment responses in patients receiving radiotherapy before and after tyrosine kinase inhibitor usage. [file 12885_2019_6140_MOESM2_ESM.docx]

**Table S1.** Comparison of treatment responses in patients receiving radiotherapy before and after tyrosine kinase inhibitor usage

|  | **Radiotherapy before TKI usage (N=12)** | **Radiotherapy after TKI usage (N=26)** |  |
| --- | --- | --- | --- |
| **Duration between radiotherapy and TKI (mean, range)** | 12.5 days (3-32) | 11.6 days (0-102)  *102d: wait for NHI SRS application |  |
| **PFS (95% CI)** | 11.4 months (4.00 to 23.5) | 13.3 months (10.10 to 36.20) | *p* = 0.938 |
| **OS (95% CI)** | 20.3 months (13.20 to 53.50) | 33.0 months (27.10 to 41.00) | *p* = 0.623 |
