# Supplementary material for: Clinical factors associated with treatment outcomes in EGFR mutant non-small cell lung cancer patients with brain metastases: a case-control observational study
Source: BMC Cancer. 2019 Oct 26;19:1006. doi: 10.1186/s12885-019-6140-0 (PMC6815404; doi:10.1186/s12885-019-6140-0)
Supplement: Supplementary file 3 — Additional file 3: Table S2. Comparison of surgery responses in patients receiving surgery before and after tyrosine kinase inhibitor usage. *The median overall survival (OS) could not be computed. [file 12885_2019_6140_MOESM3_ESM.docx]

**Table S2.** Comparison of surgery responses in patients receiving surgery before and after tyrosine kinase inhibitor usage

|  | **Surgery before TKI usage**  **(N=6)** | **Surgery after TKI usage**  **(N=2)** |  |
| --- | --- | --- | --- |
| **Duration between surgery and TKI (mean, range)** | 18.3 days (7-27) | 48.5 days (48-49) |  |
| **PFS (95% CI)** | 13.3 months (12.10 to 17.40) | 5.6 months (5.60 to 7.50) | *p* = 0.004 |
| **OS (95% CI)** | NA* | NA* |  |

*The median overall survival (OS) could not be computed.
